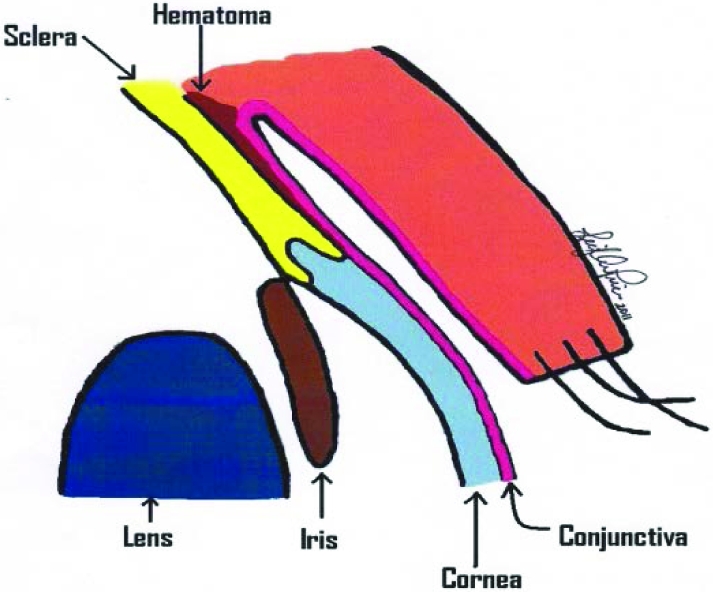# A Shot in the Face

**Published:** 2011-09-15

**Authors:** Leigh Ann Price, Justin Klaff, Emile Brown, Stephen M. Milner

**Affiliations:** Department of Plastic and Reconstructive Surgery, Johns Hopkins School of Medicine, Baltimore, MD

## DESCRIPTION

An 11-year-old boy was shot with an air-powered rifle.

## QUESTIONS

1. **Where is the entry wound?**
2. **What type of hemorrhage is present?**
3. **What are the indications for removal of the foreign body?**
4. **What is the optimal surgical approach?**

## DISCUSSION

**Figure 1:**
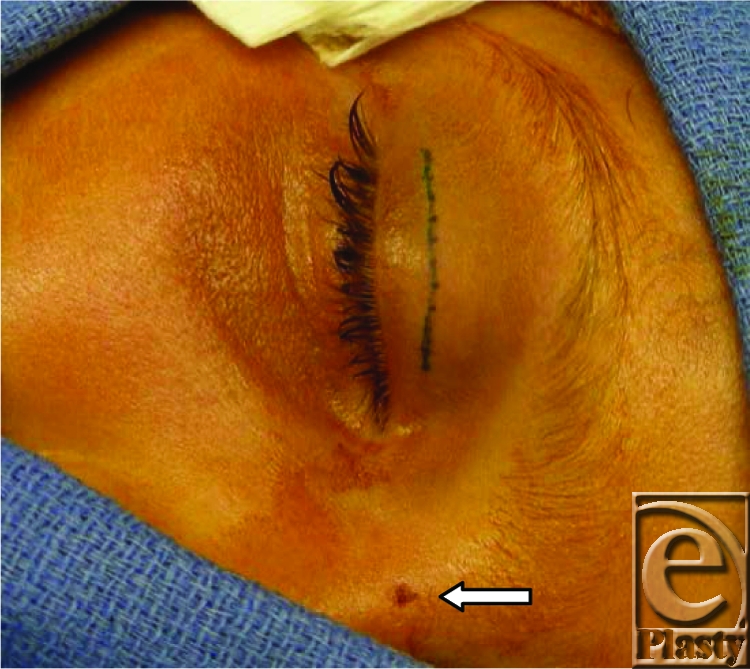


The patient is an 11-year-old boy who was accidentally shot in the face with an air-powered rifle. The ball bullet (BB) entered through the dorsum of the nose over the nasal radix (Fig 1, arrow). Radiological studies are critical to determine the position of any intraorbital foreign body.^1^

The BB traversed the soft tissue causing a subconjunctival hemorrhage (Fig 2). This type of hemorrhage is also known as *hyposphagma*, defined as an accumulation of blood trapped between the conjunctiva and the sclera. Many small, fragile blood vessels located within the conjunctiva are easily ruptured.^2^ This type of hemorrhage is usually traumatic or spontaneous and is often a self-limited condition, requiring no treatment.

Surgical removal of ballistic fragments is usually discouraged, because the dissection necessary to retrieve them can be harmful.^3^ In this case, removal is advisable. Spontaneous extrusion of the foreign body through the thin and delicate tissue of the outer surface of the eyelid can cause a cosmetic deformity. Deep extrusion can ulcerate the cornea.

The foreign body was excised using an upper lid blepharoplasty incision and can be expected to leave an aesthetically acceptable scar.

## Figures and Tables

**Figure:**
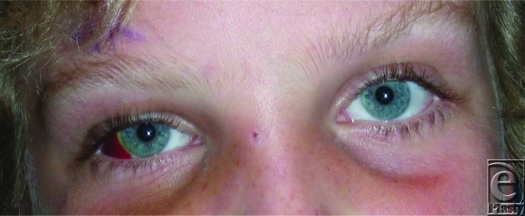


**Figure:**
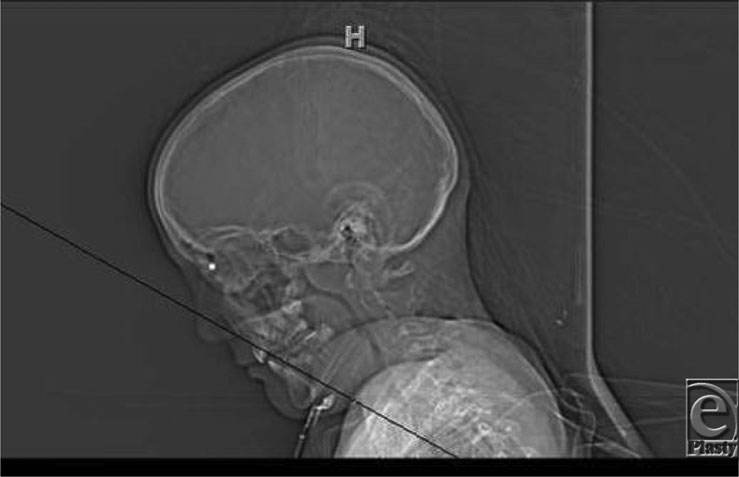

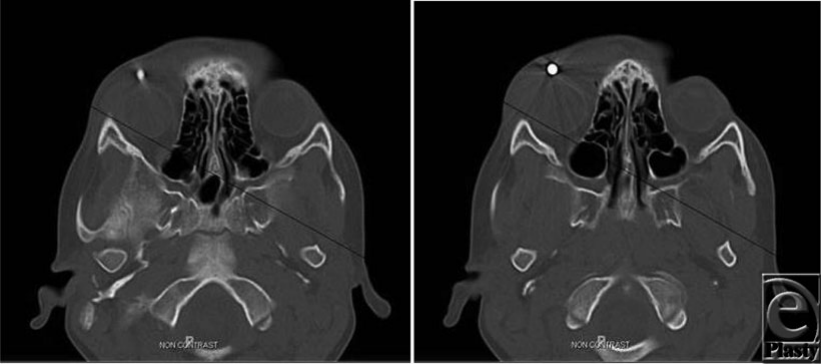


**Figure 2:**